# Contraceptive and sexual health services during the COVID-19 pandemic and recovery: a mixed-methods study in England

**DOI:** 10.1186/s12978-025-02184-x

**Published:** 2026-01-30

**Authors:** Alexandra Sawyer, Catherine Aicken, Jörg W. Huber, Jaime Vera, Deborah Williams, Moazzam Ali, Gabriela Garcia-Camacho, Armando Humberto Seuc, Nigel Sherriff, The WHO HRP Social Science Research Team, Luis Bahamondes, Jose Guilherme Cecatti, Vilma Zotareli, Rachel E Soeiro, Karayna G Fernandes, Mariana B Rogerio, Samira M Haddad, Silvana F Bento, Karla S Padua, Aline Munezero, Charles M Charles, Montas Laporte, Eunice Chomi, Seni Kouanda, Flore Marie Gisèle Donessouné, Armel Sogo, Kun Tang, Yueping Guo, Hanxiyue Zhang, Yifan Zhu, Ge Yang,, Chunxiao Peng, Xizhuo Xie, Hao Wang, Deda Ogum Alangea, Kwasi Torpey, Emefa Judith Modey, Adom Manu, Ernest T. Maya, Rozina Karmaliani, Laila Ladak, Arusa Lakhani, Marina Baig, Yasmin Parpio, Salima Somani, Pisake Lumbiganon, Jen Sothornwit, Nampet Jampathong, Somporn Rungreangkulkij, Marleen Temmerman, Ferdinand Okwaro, Abdu Mohiddin, Massimo Mirandola, Maddalena Cordioli, Alessia Savoldi, Simone Garzon, Stefano Uccella, Ranieri Poli, Nigel Sherriff, Alexandra Sawyer, Catherine Aicken, Jörg W Huber, Jaime Vera, Deborah Williams, Moazzam Ali, Caron Kim, Vanessa Brizuela, Hamsadvani Kuganantham, Grace Kapustianyk, Igor Toskin, Soe Soe Thwin, Anna Thorson, Joy Jerop Chebet, Hugo Gamerro Abrego, Armando Seuc, Gabriela Garcia Camacho

**Affiliations:** 1 School of Education, Sport, and Health Sciences, University of Brighton, Brighton, BN1 9PH England; 2 University Hospitals Sussex NHS Foundation Trust, CIRU Second Floor, Sussex House, Royal Sussex County Hospital, Brighton, BN2 5BE England; 3 Department of Sexual and Reproductive Health and Research (includes the UNDP/UNFPA/UNICEF/WHO/World Bank Special Programme of Research, Development and Research Training in Human Reproduction [HRP]), World Health Organization, Geneva, Switzerland

**Keywords:** Sexual health, STI, Contraception, Mixed-methods, Health systems, COVID-19, HIV testing, Pandemic preparedness, Access to care

## Abstract

**Background:**

Sexual and reproductive health (SRH) is essential for public health. COVID-19 led to major disruptions in the provision of essential services including SRH services. Within the context of a multi-country project, this study aimed to explore individual and service-level impacts on contraceptive and sexual health services during the COVID-19 pandemic and recovery phase in England.

**Methods:**

A longitudinal, mixed-methods design was implemented, collecting data in two phases, approximately 9 months apart (November 2021 and July 2022). The study comprised in-depth interviews with staff (*n =* 4) and clients (*n =* 20) of a sexual health and contraceptive clinical service in the Southeast of England. Over the same timeframe, a quantitative service availability and readiness assessment (SARA) was completed, based on World Health Organization validated tools.

**Results:**

Sexual health and contraceptive services continued to operate throughout the pandemic, however measures taken to prevent COVID-19 transmission and staff capacity issues (due to staff redeployment, staff sickness) impacted on patient choice (e.g. how the service could be accessed, methods of contraception available) and patient experience (e.g. delays in accessing healthcare). Despite disruptions, staff described how in-person provision remained available almost continuously for urgent/vulnerable cases. SARA data confirmed service availability, and qualitative data indicate how this was managed. For example, postal home self-sampling for STIs/HIV was expanded and contraceptive counselling by telephone was introduced to reduce clinic visits, and was retained due to popularity. At Time 2, services were running close to normal.

**Conclusions:**

COVID-19 disrupted sexual health and contraceptive services in England. Compared to pre-pandemic, more elements of these services were delivered remotely. Readiness to adapt was aided by the pre-pandemic direction-of-travel towards greater use of digital and telemedicine services. Innovations require robust evaluation to ensure optimisation for public health benefit both in the pandemic and post-pandemic context.

## Abstract (plain language summary)

COVID-19 led to huge demands on health services around the world. It caused major disruptions to sexual and reproductive health (SRH) services, the delivery of which was adapted to enable service continuity. This study was part of a global project which aimed to explore the impact of COVID-19 on SRH services. The study took place in the Southeast of England; in this paper we focused on a sexual health and contraception service. We carried out interviews and administered questionnaires at two different times, 9 months apart. We spoke to people who used this service and healthcare professionals. The service continued to run throughout COVID-19 and most people interviewed experienced minimal barriers to care. However, COVID-19 impacted the delivery, availability, and quality of services. Measures taken to prevent COVID-19 transmission and staffing issues impacted on patient choice and experience. At Time 2, services were running close to normal. However, other health service delivery and public health issues (such as mpox) impacted how the service was run. The pandemic occurred at a time when the service was already offering (or close to offering) remote elements of care. This meant that the service was able to rapidly expand remote services early on in the pandemic, such as STI home self-sampling kits. Delays in access to sexual health and contraceptive care can have serious individual and public health impacts, and our findings illustrate how these services can flexibly adapt in order to maintain continuity of these essential services during health emergencies.

## Background

In the UK, the first cases of COVID-19 were reported at the end of January 2020 [1] and community transmission was reported a few weeks later. On 23 March 2020, a national lockdown was imposed by the UK government, mandating several measures through law, such as closing of schools and non-essential shops and requiring members of the public to stay at home. This lasted until May 2020. Two further England-wide lockdowns were implemented, in November 2020 and in January 2021.^1^ On 21 February 2022, the Prime Minister set out England’s ‘Living with COVID-19’ strategy [2] with all remaining legal restrictions ending on 24 February 2022, including the legal requirement to self-isolate after a positive test. From 1 April 2022, free testing ended for most people.

COVID-19 led to huge demands on health services and to major disruptions in sexual and reproductive health (SRH) services [3, 4], both globally and in England. Disruptions to sexual health and contraception services can have significant individual and public health impacts including unintended pregnancies and undiagnosed STIs, the latter risking onward transmission and complications of long-term infection. Prompted by the pandemic, UK professional associations and organisations responded rapidly, producing guidelines, consensus statements and practice recommendations regarding the provision of SRH services during COVID-19 (e.g. British Association for Sexual Health and HIV [BASHH], Faculty of Sexual and Reproductive Health [FSRH]). For example, the FSRH identified essential SRH services at the start of the pandemic (24 March 2020) and suggested services that could be managed remotely and/or changed during the pandemic [5]. It temporarily (until November 2021) supported the extended use of some long-acting reversible contraceptive (LARC) methods, e.g. Nexplanon®, Mirena®, Levosert®, and 10-year copper IUDs [6]. The guidelines also advocated the use of the progestogen-only pill (POP) as a ‘bridging’ method for contraception (a short-term supply of POP to bridge the gap between emergency contraception (EC) and longer-term contraception).

### Objectives

The aim of the current study was to understand individual and service-level impacts on contraceptive and sexual health services in England during the COVID-19 pandemic and recovery.^2^

## Methods

### Design

This longitudinal, mixed-methods study collected data in two phases, approximately 9 months apart. This enabled comparison of service delivery, provision, access, and use over time as the pandemic progressed. This study took place within a wider, international study on the pandemic’s impacts on SRH provision, however as the current study focuses on England, we make no comparisons with findings from other countries.

### Study setting

The study setting was a Sexual Health and Contraceptive Service in a city in the Southeast of England. The service operates clinics at three sites and provides a comprehensive sexual health and contraceptive service for the general population, including dedicated clinics for key populations. Its sexual health service includes symptomatic and routine testing, treatment and partner notification (contact tracing) for sexually transmitted infections (STIs) including HIV (in clinic or via home self-sampling kits where appropriate) and pre-exposure prophylaxis (PrEP). In the UK, including England, these services are provided for free at the point of use, and no prescription charges apply for treatment or contraception.

### Qualitative component

Data collection comprised qualitative, semi-structured in-depth interviews (IDIs) with staff and clients. Topic guides were developed based on the global study’s master protocol [7] and enabled individual-level impacts on clients and healthcare professionals (HCPs) to be explored, alongside health facility level impacts, by exploring HCPs’ perceptions of the availability and readiness of their health facility to continue to provide services during the pandemic. The topic guides were adapted to the English context and local service configuration by the research team and pre-tested.

Data collection commenced in Autumn 2021 (Time 1; November 2021 to April 2022) and the second phase began in Summer 2022 (Time 2; July to November 2022). Time 1 data collection partly coincided with the Omicron wave of COVID-19 which caused significant pressure on health services.

### Participants and recruitment

#### Healthcare professionals (HCPs)

We aimed to interview the same 3 HCPs at Time 1 and Time 2 to gain their reflections on changes over time within the service. The facility manager took the role of ‘gatekeeper’ and approached eligible colleagues to ask if they were interested in participating.

#### Clients

We sought to interview 10 clients at Time 1 and 10 different clients at Time 2. Clients were approached by staff at the facilities who acted as ‘gatekeepers’.

### Data collection

Interviews were scheduled at participants’ convenience and took place online (e.g. Teams) or by telephone. A brief questionnaire was administered to capture participant characteristics. Clients were offered a £10 voucher as a token of thanks for their participation. HCPs were not offered a voucher.

### Data analysis

Audio-recordings of IDIs were professionally transcribed. HCP and client transcripts were analysed as two separate datasets. We used qualitative Content Analysis, a stage-based approach to analysis, and analysis was descriptive. Use of NVivo and the Framework process for data management allowed sharing of the coding and analysis process within the study team, and therefore transparency and rigour of the analysis. Comparisons over time were explored as part of the analysis.

### Quantitative component

#### Questionnaire

To understand how health facilities delivered SRH services during the emergency response to COVID-19 (the wider, international study’s objective), a quantitative assessment of service availability and readiness was completed, involving a health facility questionnaire. This questionnaire was developed based on the following WHO validated tools [8–10]: (i) Service Availability and Readiness Assessment (SARA) guide; (ii) health emergency facility assessment checklist in emergencies; (iii) safe abortion assessment tool; (iv) gender-based violence health system readiness tool; and (v) STI assessment tool. The questionnaire included indicators of the availability and readiness of: (i) Policies and plans; (ii) Services maintenance and referrals; (iii) Infrastructure; (iv) Commodities; and (v) Human resources. The questionnaire contained a general health services continuation section as well as sections pertaining to the specific SRH service (in this instance, contraceptive and sexual health services, including HIV). As the questionnaire was adapted from various existing tools, it was reviewed by local project collaborators and minor adjustments were made. In the study reported in this manuscript, the questionnaire was completed by a senior HCP from the sexual health and contraceptive service.

#### Timing

The health facility assessment modules were implemented during November 2021 (Time 1; baseline) and approximately 9 months later, July 2022 (Time 2; endline).

#### Data analysis

Descriptive analysis was used to illustrate the basic characteristics of the facility, including the monthly number of clients, types of services, procedures, items and medicines provided, number of medical staff, stocks of essential medicines and supplies. Tentative comparisons were made between Time 1 and Time 2. It was not possible to conduct an in-depth data analysis at a local level as the limited data points restrict us from drawing conclusions of statistical significance.

## Results

### Qualitative interviews with HCPs and clients

#### Sample description

##### Clients

Table 1 describes the 20 client participants recruited from the sexual health and contraceptive service. Their ages ranged from 21 to 69 (*M* = 36.7). Clients described accessing the clinic for diverse sexual health and contraceptive needs including STI/HIV testing (routine or symptomatic) and STI treatment, for pre-exposure prophylaxis (PrEP), contraception (including EC, LARC insertion [to prevent pregnancy, manage menopausal symptoms] and removal), cervical cancer screening, treatment of other genito-urinary syndromes and for the mpox vaccine. They sometimes described receiving STI testing and prevention when accessing the service for other reasons. At both timepoints most clients in our sample reported having accessed the service more than once during COVID-19, with some having accessed the service regularly (either remotely or in person) and as frequently as every three months. The main reasons for regular use were access to PrEP and/or STI testing. Only one person (interviewed at Time 2) reported their current visit to the clinic, which was for the mpox vaccine, as their first time ever accessing the clinic. Therefore, most participants were able to reflect on earlier experiences of accessing the service during COVID-19, as well as pre-COVID-19.

**Table 1:** Client participant characteristics

Characteristic	(*N =* 20)
Gender	
Male	9
Female	11
Sexual orientation	
Heterosexual	7
Gay man	9
Bisexual	3
Pansexual	1
Relationship status	
Single	9
Living together (unmarried)	1
Partner (not living together)	4
Married	5
Polyamorous relationship	1
Ethnicity	
White British	11
White Other	5
Mixed	2
Arab	1
Latin American	1
Religion	
None (or agnostic)	14
Christian	5
Other—spiritualist	1
Education	
GCSEs/O-Levels/school until 16	2
A-Levels/equivalent/school until 18	6
Undergraduate degree	6
Postgraduate degree	6
Employment	
Part-time employment	2
Full-time employment	9
Self-employed	2
Unemployed	1
Retired	3
Full-time student	3
Number of children	
0	16
1	0
2	4

##### HCPs

Three HCPs were interviewed at Time 1 and one at Time 2 (*n =* 4). Roles of participants included: sexual health adviser (SHA),^3^ associate specialist in contraception and sexual health, and lead nurse.

#### HCPs’ perspectives and experiences

##### Health facility availability and readiness

The section below summarises the impact of COVID-19 on health facility availability and readiness to provide sexual health and contraception services, from the perspectives of the HCPs interviewed. 

##### Facility mobilisation

The sexual health and contraception service was not designated a COVID-19 treatment, testing or vaccination facility. 

##### Service delivery

Staff noted that clients’ sexual contact with people outside the household did not stop during lockdowns and recognised that provision of contraception and STI/HIV testing/treatment were essential services. Whilst these services continued to be provided, capacity was stretched at times and patient choice was impacted. A long period of frequent changes to service delivery was described. During the first lockdown, the clinic reduced its service provision to only the most urgent acute cases which could not be managed remotely. For example, people who had been sexually assaulted, and those in need of Post-Exposure Prophylaxis (PEP) or emergency IUD came into the clinic. Although staff would *“do as much as we could on the phone”*, they had *“discretion if we thought someone was vulnerable but didn't come under a set heading”* to see them face-to-face in clinic. Clients with STI symptoms were provided with treatment by post where feasible, based on presumed rather than confirmed diagnosis, and communications technology was used to support this (e.g. telephone consultations; use of a secure email address for sending photos). Contraceptive pills and patches were provided by post, and LARC provision was suspended only during the first lockdown. Telephone triage was used to determine whether care could be provided without a client needing to come into the clinic, and this has remained in place. Before the pandemic, home STI self-sampling was already provided by the clinic for patients not reporting symptoms (where the client orders a sampling kit online, provides samples, and mails it back for lab-based testing) and this service remained available. Early in the pandemic the postal self-sampling service was extended to patients reporting certain symptoms, who would normally be asked to attend clinic.

Service delivery went through several iterations, with gradual increased provision of services in clinic. Although in-person service provision reduced again in the second and third lockdowns, this was to a lesser extent than during the first lockdown. For example, routine vaccination for Hepatitis B (offered to at-risk groups) continued during the third lockdown; LARC provision was only suspended during the first lockdown. Over time there were changes in what services were provided without seeing a client in person – for example, an early change was that symptomatic patients were once again seen in clinic, where they could be examined and microscopy could be undertaken. At Time 1, the usual ‘walk-in’ clinic sessions which were suspended during the first lockdown had still not resumed. Instead, booked appointments and restricting patients from bringing a partner/friend (who was not themselves a patient) gave the service greater ability to manage the number of people in the waiting area.

When conducting consultations by phone, some staff reported concerns about missing signs of other problems (e.g. emotional distress, domestic violence or coercion) because of not being able to read the client’s body language, and concerns about patient confidentiality.

*One did feel that one might be missing things, especially in vulnerable patients – you never quite know what’s going on in the household when you’re speaking to somebody, and if there's any coercion or anything going on in the background and you don’t get any visual clues when you’ve got somebody on the phone (Time 1, Contraception Specialist)*

Text messaging was offered for some consultations, with the rationale of providing a discreet communication channel for young people who were at home with their family. Telephone consultations for contraception counselling – introduced during the pandemic – were popular with clients. At Time 1, almost all consultations were provided in person as before the pandemic (a few telephone slots were kept for people who could not attend the clinic), except for contraception consultations which were still offered by phone or in person. Where clients should have their blood pressure checked before receiving hormonal contraception, this was done when they attend clinic to collect their contraceptive supplies following a telephone consultation.

*A positive thing that came out of it, is that lots of people really like to access contraception in that way [receiving contraceptive supplies following a remote consultation], and it is actually a really easy way to access contraception. So, we have continued a Virtual Contraception clinic for women who want to get more supplies of their birth control, whether that be pills or patches (Time 1, Lead Nurse)*

Partner notification was managed as before (pre-pandemic), and the clinic already used the SXT service (an online text message and email service providing sexual health information and decision support, and supporting partner notification). The option to send pictures to clinicians was available, which did not exist before the pandemic. Though not used as much as it was earlier in the pandemic, symptomatic patients (at Time 2) who contacted the clinic were offered this facility, and based on photographs the clinician decided whether a visible symptom, such as a rash or a lump, necessitated clinic attendance.

Staff considered that some of the most vulnerable people (such as people who do not speak English, people experiencing mental health challenges, and those with chaotic lifestyles) may have found it particularly difficult to access the service. By Time 2, availability of the clinic’s drop-in services had not returned to pre-pandemic levels, and it was reported that when the service was stretched, the outreach drop-in service located near some local schools – which had only just resumed - was the first to be suspended. This was thought to affect access for vulnerable groups including young people.

Service delivery was also affected by (i) changes in other areas of the health system, (ii) capacity issues over winter 2021/22, and (iii) mpox. First, the clinics provide contraceptive services which are also provided by primary care (general practice) and receive and make referrals to other services. Primary care services were overstretched, and reduced or ceased provision of LARC methods, specifically IUDs and implants which must be fitted by healthcare professionals with up-to-date certification of clinical competence. This, together with referrals received from other services (including sexual assault referral centres and abortion services) meant that locally, for some time since the first lockdown, the clinic experienced much increased demand for these methods, which was still evident at Time 2. The sexual health adviser described difficulties in managing onward referrals for mental health needs, with challenges in finding out which services were running and how they were accessed. It seemed as if clients “*absolutely had to be in dire crisis” *before being able to access support. Second, staffing, and therefore capacity, was strongly impacted by COVID-19 illness and self-isolation in winter 2021/22, reflecting the global emergence of the omicron variant. Third, as an STI testing and treatment service, capacity was impacted by mpox which a staff member at Time 2 estimated took up to 20–30% of clinic workload in Summer 2022 (when there was no mpox vaccine). Clients suspected to have mpox had a telephone consultation and attended a dedicated room in one clinic which was deep-cleaned between each patient, up to an estimated six patients daily. This took up almost all of one clinician’s time and that of another person, meaning the rest of the clinic’s activities were*‘concertinaed’ *into the remaining space and capacity. 

#### Workforce (human resources)

##### Redeployment and work elsewhere in the health service

At the start of the pandemic the ‘vast majority’ of the service’s team were redeployed, within the same hospital Trust. They reportedly felt poorly prepared for their roles on acute wards, which were very different from their work at the sexual health and contraception (outpatient) service, and psychologically very challenging. Though they received some training this was perceived as limited, they often lacked recent acute care experience and were isolated from familiar colleagues. Redeployed staff returned gradually. One manager described advocating for the return of staff who were distressed with their redeployment roles – both to protect colleagues, and to increase the capacity of the sexual health and contraception service.

*I think in the first wave a lot of staff went over and had really very little support from other members of staff, and found that very difficult to be in a situation where they had no experience and then also no support, and really felt that they were unable to help anybody (Time 1, Contraception Specialist)*

##### Impact of infection, and infection control measures, on staffing

Further effects on staffing related to measures to protect those most at risk from COVID-19 illness, and to prevent transmission. Early in the pandemic, staff deemed ‘clinically vulnerable’ stayed away from the facilities to avoid exposure, with some working remotely. Interviewees described how, early in the pandemic, some colleagues limited their risk of transmitting COVID-19 to family by staying away from home, or by no longer providing care in person to older relatives. National guidelines, which changed over time, initially recommended self-isolation when symptomatic, after a positive lateral flow or PCR test, and following contact with someone with confirmed or suspected COVID-19. This impacted on staffing levels, and early in the pandemic services sought to minimise contact between staff. Staff reported how they and most of their colleagues were vaccinated shortly after vaccination became available in England (vaccination of frontline health workers was prioritised across the country).

##### Adapting to changes in service delivery

Staff had to adapt rapidly to changes in the delivery of the services they provided, with little or no formal training. Staff felt they were dealing with constant changes to service delivery, which took place over a long period at the service. Changes to practice seemed to be most readily accepted where their rationale was clear to staff – for example, safety for staff and clients, or maintaining service continuity and access. However, learning new ways of delivering services (e.g. switching to telephone consultations) was time-consuming, and this reduced productivity, at least initially.

##### Information systems (reporting systems)

England had national reporting systems in place for reporting COVID-19 test results and contact tracing. The hospital trust had its own additional reporting systems, which appeared to be used for COVID-19 sickness absence monitoring, for measuring COVID-19’s impact on the service and consequently informing service planning. Staff reported receiving summaries of this information, via regular email bulletins, which also indicated current COVID-19 guidance. Staff reported eventually becoming fatigued by this information and no longer reading it every time. These communications eventually reduced in frequency or ceased. As an interviewee reported, by mid-2022, COVID-19 sickness was reported using the ordinary sickness absence reporting system, without further specification.

##### Access to essential medicines and supplies

Staff did not report a prolonged lack of essential medicines, personal protective equipment (PPE) or other essential supplies, although some mentioned shortages and delays, even at Time 2. These were managed by offering patients alternative medications or shorter supplies. Staff were not always aware of the cause of any delays in replenishing essential medicines or supplies, and whether this issue was related to a local issue (e.g. distribution problem within the hospital, perhaps related to staffing). In addition to the indirect and direct effects of the pandemic, staff also mentioned Brexit (the UK’s departure from the European Union, which influenced trade), and the March 2021 Suez Canal blockage, as possible reasons for disruptions to supply.

##### Leadership and governance

Staff reported that national guidance was followed. Where they went into further detail, this referred to the national guidance from Public Health England and NHS England. The relevant professional organisations (e.g. FSRH, BASHH, RCOG) provided guidance early in the pandemic as well, as did the clinic’s hospital Trust.

#### Clients

Experiences of accessing sexual health and contraception services (from the client perspective) during COVID-19 are presented in four thematic areas^4^: (i) initial information seeking; (ii) client journey – process of seeking and receiving care; (iii) perception of risk of COVID-19 infection during care-seeking; and (iv) barriers and facilitators to receiving care (including reasons for delays).

##### Initial information seeking

Clients accessed the service directly without going through their GP or other healthcare service/provider as the dedicated clinics were their usual point of access for their sexual health and contraceptive needs, and such services are accessible without referral in the UK. One participant went to the clinic out of *“desperation”* as primary care had not been able to help.

*I couldn’t get anything from the GP. Basically, he washed his hands of me and said you need to go privately, I can’t help you…It was desperation really, I had nowhere else to go….that’s why I contacted them [sexual health and contraception clinic] (P17_03, attending clinic regularly throughout COVID-19 for treatment of vaginitis, Time 2 – July 2022).*

Clients sought information about the service online before access and typically described this information as helpful and informative, although opening times/services changed throughout COVID-19. In contrast, at the start of the first lockdown “*it was harder to figure out how to get tests and be tested…”*, as one participant reported.

##### ‘Patient journey’ – process of seeking and receiving care

Clients reported accessing the clinic in a variety of ways including telephone consultations, in-person, and/or ordering STI self-sampling kits online. Client participants booked appointments online, over the telephone, and/or went to a walk-in clinic session (these ceased on 23 March 2020 and resumed on 6 May 2021). Participants reported being triaged for in-person appointments either when booking and/or after telephone consultations. There were mixed experiences regarding appointment availability depending on when participants were trying to access the service. The first lockdown (March to June 2020) was reported by several clients as being the most difficult time to access the clinic when in-person appointments were not widely available. Clients noted that in-person appointments became gradually more available as the pandemic progressed, with some reporting that it was even easier to get an appointment compared to pre-COVID-19. In comparison, others reported longer waiting times compared to pre-COVID-19.

*I actually had loads of contact with that clinic during the whole pandemic and I actually thought it was really good, it was better than normal, because you used to have to sit and wait. You literally just rang them, and they triaged you straightaway and then they just brought you straight in, like an hour later. It was like unreal how efficient and organised it all was (P19_03, attending clinic for sexual health screening, Time 2 – August 2022).*

Although in-person appointments were restricted, especially at the start of the pandemic, clients tended to be seen in-person promptly if they were symptomatic or needed time sensitive care and/or treatment. For example, the two participants who required EC were seen at the clinic within 48 h. Another participant reported accessing the clinic in-person every fortnight since October 2020 because her condition requires regular monitoring and treatment. As the pandemic progressed more participants reported being able to access in-person appointments and walk-in clinics reopened. One participant who accessed the walk-in clinic in June 2022 observed that the service seemed back to normal.

*I’d say it’s filtered back down into as near as it was before. I guess it’s kind of smoothed out. (P11_03, regular attendance at clinic for STI testing, Time 2 – June 2022)*

Clients who accessed STI testing in-person generally reported that results were received promptly. However, one client who accessed the clinic in July 2022 and was subsequently diagnosed with mpox was still waiting after three weeks for the results of STI tests. Participants were largely understanding about the impact of COVID-19 on limited availability of in-person appointments and/or waiting longer for test results. However, this did not fully mitigate the frustration and anxiety felt by some of the participants.

*It was unsettling and uncomfortable, because at the time I did contract something and I did need to see someone. This was 2021 I think when I think there was another lockdown…The longer I left it the worse it got, it took hold quite quickly. I was a little bit unsettled by it and just left in the dark really. It was hard to get hold of anyone to get some advice, and even get that appointment and go on and see someone. (P11_03, regular attendance at clinic for STI testing, Time 2 – June 2022).*

Throughout the pandemic many clients reported either ordering self-sampling kits online which were then posted to them or collecting kits directly from the clinic or pharmacy. Clients who tried to order self-sampling kits but were symptomatic were encouraged to book an appointment with a HCP. Postal self-sampling kits arrived promptly. Participants reported mixed experiences with sampling kits such as difficulty taking a blood sample. Clients typically reported waiting a couple of weeks for results after using self-sampling kits, although sometimes a little longer and one client reported waiting two months for results, which came back as inconclusive.

*It was probably the most difficult experience I’ve had at a sexual health clinic, because of the delay, you know, the waiting…And then, not knowing what’s happening and try and call up and not getting through. And I guess maybe the fact that I couldn't access in person made it more difficult to find out what was happening. (P02_03, regular attendance at clinic for STI testing and PreP, Time 1 – November 2021).*

Finally, clients were positive about the staff and service describing it as *“non-judgemental”, “lovely”, “really nice”,* and *“reassuring”.*

##### Perception of risk of COVID-19 infection during care-seeking

Most clients did not consider the risk of COVID-19 infection when accessing sexual health and/or contraception services at the clinic. Those who did consider this were not concerned about it because they felt that provisions were in place to minimise risk. One participant who accessed the clinic in June 2022 (Time 2) described how COVID-19 was no longer a consideration or concern for her.

*I was never worried about COVID, never came into my mind as a risk factor when accessing them [appointments at the clinic]. (P02_03, regular attendance at clinic for STI testing and PreP, Time 1 – November 2021).*

For the small number of clients who did consider the risk of COVID-19 infection when making the decision to seek sexual health/STI and contraception services, the need to access timely testing, treatment, and/or contraception surpassed the fear of catching COVID-19. For example, one participant who had been identified as clinically extremely vulnerable^5^ because of their cancer diagnosis and treatment described feeling *“petrified”* of being infected with COVID-19 throughout the pandemic. However, at the point of her first access at the clinic (October 2020) her symptoms and need for treatment were so pronounced that the fear of catching COVID-19 was no longer a concern. Instead, her main worry was that appointments might be cancelled by the clinic because of COVID-19 related reasons.

*I was probably more worried about the appointment being cancelled or them having all gone down with COVID so no one could see me. That’s probably more what I was worried about than thinking I might catch COVID. I just wanted to desperately be seen. (P17_03, attending clinic for treatment of vaginitis, Time 2 – July 2022).*

All clients described a range of COVID-19 infection protection and prevention measures implemented at the clinic(s) including mask wearing by clients and staff, hand sanitiser, spacing between chairs in the waiting area, temperature checking at reception, and a screen at reception. The implementation of these measures did not negatively impact on care experiences, however one participant noted that answering personal questions about sexual activity was more challenging with a facemask. Finally, several clients at Time 2 noted that some COVID-19 safety measures (such as mask wearing by clients in the waiting room and/or hand sanitising) were either not enforced or not as rigorously enforced compared to their experiences of attending the clinic earlier in the pandemic. One participant commented that no restrictions were in place at their last visit to the clinic (June 2022).

*Upon my visit I was quite disappointed by the fact there are posters everywhere saying please wear a surgical mask and probably only 20% of people visiting were. (P20_03, accessed clinic for mpox vaccine, Time 2 – October 2022).*

##### Barriers and facilitators to receiving care

Clients generally thought remote consultations via telephone improved access to care, especially during the early stages of the pandemic when in-person appointments were limited. Some participants also noted that triaging ensured in-person appointments were given to those who needed to be seen, which reduced waiting times (both for an appointment and when at the clinic). The replacement of walk-in clinics with triage and appointment systems was viewed positively by one participant as it was easier to plan their visit around other commitments.

*It's always been walk-in services, and I found them a lot harder, and a bit of a barrier to me accessing them, compared to the appointment (P05_03, regularly attending clinic for STI testing and PrEP, Time 1 – February 2022).*

However, two clients described their struggle with being diagnosed and treated on the basis of remote consultations.

*In the first lockdown I found it very difficult because I really wanted to go into the clinic, because I was having a lot of issues…And, I was getting nowhere with the GP, and because you couldn’t be actually looked at, you had to send pictures of your symptoms and I found that extremely unhelpful. (P03_03, regular attendance at clinic for STI testing, Time 1 – December 2021).*

Self-sampling tests for STIs were perceived as both a facilitator and barrier to accessing care. Some clients strongly preferred self-sampling kits (compared to accessing a clinic to be tested) and others found aspects of self-sampling challenging. Clients commented that self-sampling made STI testing more accessible during COVID-19, and even when clinics provided in-person appointments the convenience of postal tests meant they did not have to travel to the clinic. Greater accessibility and reduced barriers (e.g. waiting at clinic, taking time off work) to accessing sexual health care were highly valued by clients.

*Much, much easier [postal tests], I’ve always wondered why they don’t do postal tests. I’ve always thought about the number of people that can’t go and wait from eight to five on a Friday, but especially people who are not symptomatic, why would they ever take a day off work to do that. (P19_03, attending clinic for sexual health screening, Time 2 – August 2022).*

When using self-sampling kits, participants reported most difficulty with obtaining a blood sample using a fingerprick, finding it painful and/or not being able to collect enough blood. These challenges meant that several participants reported either abandoning the kit or getting an invalid test result due to an insufficient sample.

*I found it difficult, particularly doing a blood test, I found it difficult at home. It doesn’t seem to work for me, and so I get lots of pricks all over my finger but no blood. (P02_03, regular attendance at clinic for STI testing and PreP, Time 1 – November 2021).*

One participant reported feeling overwhelmed by the instructions and therefore delayed testing. Several participants thought that STI testing in clinic was more appropriate as HCPs can provide more information about the test and results (for example, one participant was uncertain of the best time to test for STIs after unprotected sex). Several participants also had concerns about the accuracy of results obtained from self-sampling kits.

*When it comes to STIs, it's such a worrying kind of notion that you have to be tested for things like that. And, I think having someone go over things with you whilst that's being done, I think it's much easier (P03_03, regular attendance at clinic for STI testing, Time 1 – December 2021).*

Whilst most clients were able to access the sexual health and contraception care they needed, appointments for removal of LARC were comparatively more challenging, with two participants being told they could not have their LARC removed. This left one participant feeling frustrated and unheard, and they considered accessing private healthcare. In comparison, fitting of an IUD for (emergency) contraception or as menopausal symptom management was more easily accessible with in-person appointments provided more promptly. Finally, concern about being seen to break COVID-19 rules by being sexually active when contact between households was restricted was viewed as a barrier in seeking healthcare by two of the participants at the start of the pandemic. One participant described how they had to *“work harder to get STI tested”:*

*At the beginning it did feel like they didn’t want you to come in and it did feel like they were telling you, you shouldn’t be having sex with anyone, you should be isolating, so you shouldn’t have to come into the clinic (P10_03, regular user of STI self-sampling kits, in-person STI testing, & PreP Time 1 – April 2022).*

#### Findings from the service availability and readiness assessment

##### Health services continuation module

*Policies and plans –* There was a national SRH essential health package prior to COVID-19 and a nationally identified core set of essential health services to be maintained during COVID-19 at both timepoints. There was additional government funding allocated to provision of essential health services. 

##### Health services continuation module

*Policies and plans –* There was a national SRH essential health package prior to COVID-19 and a nationally identified core set of essential health services to be maintained during COVID-19 at both timepoints. There was additional government funding allocated to provision of essential health services.

*Maintenance of essential health services –* At Time 1 government policies regarding outpatient services, inpatient services, community-based care, and mobile clinics were to run a limited service, but emergency unit services and prehospital emergency services were to function as normal. At Time 2, government policies were for all services to function as normal. At Time 1 most health services were disrupted due to COVID-19, however at Time 2, responses indicated that no health services were disrupted apart from accident and emergency care, which was noted as partially disrupted. A range of approaches were being used to overcome the disruption to essential health services in public sector health facilities including: telemedicine, task shifting/role delegation, novel supply chain and/or dispensing approaches for medicines through other channels, triaging of priorities, and redirection of patients to alternative healthcare facilities*.*

##### Contraception module

*Services and referrals –* National contraceptive care guidelines and checklists/job aids were available in the facility. Between May 2021 and October 2021 (baseline) an average of 740 clients used the service per month for contraceptive counselling and/or services; at endline (between January 2022 and June 2022) an average of 741 clients used the service per month.

*Infrastructure –* Indicators relating to facility infrastructure suggest these were available at both timepoints (e.g. convenient opening hours, reception desk at facility, private counselling/examination rooms, written information and materials available for clients to take home and read).

*Commodities –* At both timepoints no medicines or commodities (i.e. different contraception methods) were out of stock.

*Human resources –* Staff had received training in family planning in the last six months, as well as training in adolescent SRH. At both timepoints there were approximately 25 staff involved contraceptive service provision.

This data indicates facility readiness and availability to provide contraceptive services during both data collection periods.

##### STI module

Between May 2021 and October 2021 (baseline) an average of 4263 clients used the service per month for STI counselling/services; at endline (between January 2022 and June 2022) an average of 4703 clients used the service per month.

*HIV—*At both timepoints, the facility offered HIV counselling and testing services; HIV rapid test kits; HIV antiretroviral prescription/treatment or treatment follow-up services; and condoms.

*STIs—*At both timepoints, the facility offered testing/treatment of STIs. HCPs had received training in STI diagnosis and treatment in the last two years.

*HIV/STI testing component and STI vaccines:* The facility offered a range of HIV/STI tests on site (e.g. syphilis rapid testing (although responses indicated that this was only available at Time 2), HIV rapid testing, syphilis dark field microscopy, urine rapid tests for pregnancy, urine protein dipstick testing, urine glucose dipstick testing, urine ketone dipstick testing). There were no shortages of drugs for STI treatment at the facility at either timepoint.

*Partner notification and contract tracing:* The facility provided and supported partner notification and contact tracing at both timepoints.

This data indicates facility readiness and availability to provide HIV/STI services during both data collection periods.

## Discussion

This study provides evidence of how COVID-19 impacted the delivery, availability, and quality of sexual health and contraception services. Although these services continued to operate throughout the pandemic, we have been able to detail how patient choice and patient experience was impacted by measures taken to prevent COVID-19 transmission and staff capacity issues. At the start of the pandemic, in-person appointments were limited to particularly vulnerable clients and to provision of urgent care/treatment which could not be provided remotely. Over time, as the pandemic progressed, in-person consultations generally increased. Our study shows how whilst the sexual health and contraceptive service was operating close to normal at Time 2, wider health service delivery (e.g. primary care limiting LARC fitting) and public health issues still impacted recovery. In the UK, sexual health clinics were at the forefront for the country’s mpox response (being responsible for testing, diagnosis, and treatment), which added significant pressure to services that were still impacted by the COVID-19 pandemic. Wider sexual health services were limited as resources were reoriented to the mpox response [11].

The pandemic occurred in a context where many UK sexual health services were already moving towards offering ‘remote’, telephone and/or online elements of service provision. The sexual health and contraception service in this study was therefore able to rapidly expand its pre-existing remote services early in the pandemic, such as postal STI self-sampling kits.

### Strengths and limitations

This mixed-methods study provides an in-depth insight into health facility availability and readiness of a sexual health and contraception service during COVID-19, including clients’ and HCPs’ experiences of access to, and delivery of, these services. The longitudinal design enabled comparison of service delivery, provision, access, and use over time as the pandemic progressed. In common with other studies that explore one service in depth, caution should be exercised in considering the transferability of our findings beyond the context in which the data were collected. However, the service in our study is large and diverse. During the data collection period, the impacts of COVID-19 on all health facilities was immense, including problems with staffing and sickness more broadly as well changes to clinical protocols bringing difficulties for HCPs and the facilities, which is a likely cause for the slightly lower recruitment of HCPs (two were not interviewed at Time 2). Limitations in our sample include limited ethnic diversity among clients (which may be because of ethnic disparities in access to care, or differential offering or uptake of the interview invitation). Our study therefore cannot contribute to understanding ethnic disparities in access to care. Study design meant that all client participants had accessed care and/or treatment (but who may have had either positive or negative experiences of this) and excludes people who were unable to access the service at all. Our consistent finding that client interviewees had little or no concern about catching COVID-19 through attending a sexual health clinic is unlikely to extend to those who may have needed sexual healthcare but did not access it (for instance, due to fear of COVID-19 exposure, lack of awareness that services were still operating, or fear of judgment for breaching social distancing rules). As HCP interviews suggest, this may include some of the most vulnerable patients, including those who are served by the severely disrupted outreach services. However, within the interview, participants were asked to reflect on earlier access to sexual health and contraceptive services (including at the start of the pandemic) and there was diversity in their experience of care-seeking and service access.

### Meaning and implications

These findings complement previous research on how COVID-19 has disrupted and impacted SRH service delivery [4, 12]. The sexual health and contraception service continued to operate throughout the pandemic and client participants that we interviewed were able to access the access to sexual health and contraceptive services they needed, indicating resilience in service delivery. Interviews with HCPs indicated that some of the most vulnerable people (e.g. people who did not speak English, people experiencing mental health challenges, young people) may have found it particularly difficult to access the service during the pandemic, a finding which has been supported by other UK studies looking at SRH access [13, 14], and which was also suggested by client interviewees who discussed experiencing great difficulty in accessing other health services. Suspension of outreach services may have also had detrimental impacts on the accessibility of SRH services during COVID-19.

The rapid implementation (and expansion) of virtual and remote healthcare that we observed in our study was one of the most significant changes within healthcare, during the COVID-19 pandemic. The sexual health and contraceptive service that our study focuses on was relatively advanced compared to other health areas and was therefore able to expand its existing provision of postal self-sampling kits and telehealth, with some benefits to the accessibility and acceptability of services. The key benefits and limitations of telehealth identified by participants align broadly with those in the literature and included safety (e.g. minimising risk of COVID-19 infection), privacy, convenience and access, offset by challenges associated with the lack of physical examinations, difficulty communicating without body language. There were also service-specific challenges with provision of remote care. The general acceptability of STI self-sampling kits, and limits to this (e.g. some people’s difficulties with taking blood samples), have been reported in other studies [15, 16]. Clients and HCPs also mentioned the missed opportunities for sexual health promotion discussions when there is no contact with a clinician, which is often the case with self-sampling (unless there is a positive result). As others have noted [17] some staff reported concerns about missing safeguarding issues because of the lack of visual cues during telephone consultations. Although HCPs’ confidence in identifying safeguarding concerns may increase as they become more experienced in delivering sexual health and contraceptive care via telemedicine [18], the importance of patient choice, and of offering in-person appointments for those with language difficulties, vulnerabilities, and/or heightened emotional distress is paramount.

Timely access to sexual health and contraceptive care is important, and whilst most of our study’s participants were able to access the service and/or treatment they needed, delays (or longer than anticipated waits) were discussed frequently by participants (clients and HCPs). Delays in timely access to SRH services in Britain during COVID-19 have been reported elsewhere [12]*.*

The pandemic led to changes in some HCPs’ responsibilities, including redeployment to acute care. Insufficient training, and insufficient consideration of the skills of redeployed staff have been widely reported in previous studies [19, 20], and this may particularly be the case for staff from sexual health and other outpatient services.

### Implications and future research

Alternatives to in-person care, such as telephone/video consultation, STI self-sampling kits (especially within services/populations where uptake and acceptability of self-sampling for STIs has not been explored) must be rigorously evaluated. Further research is needed to understand where telemedicine in relation to SRH services is beneficial and where it poses unique challenges and potentially increase healthcare inequities [4, 21]. Development of stigma mitigation strategies is important to address the perceived stigma related to having sex during the pandemic outside of household members (particularly when ‘stay at home’ restrictions are in place) as this can be a potential barrier to accessing sexual health services [22]. Information about services (such as clinic opening times, booking options) need to be easily accessible and up-to-date, especially online information as this is where most people first look to find out about the SRH services they need. Finally, there is a need for better outbreak preparedness to ensure SRH staff are trained/skilled for redeployment, especially those unused to working in acute settings.

## Conclusions

Delays in access can have serious individual and public health impacts, and the findings from this study reflect the importance of maintaining these services during health emergencies. Expansion of pre-existing remote services was implemented during the pandemic, and while some of these changes were temporary, other adaptations have become permanent with lasting impacts. Innovations require robust evaluation to ensure optimisation for public health benefit both in the pandemic and post-pandemic context.

## Data Availability

Quantitative data will be available upon request as per the WHO policies. Requests for access to data can be sent to alimoa@who.int. Excerpts of the qualitative interview data are available within the manuscript. Participants did not provide consent for interview transcripts to be made publicly available.

## Abbreviations

COVID-19: Coronavirus Disease 2019
EC: Emergency Contraception
FSRH: Faculty of Sexual and Reproductive Healthcare
HCP: Healthcare Professional
HIV: Human Immunodeficiency Virus
IDI: In-Depth Interview
IUD: Intrauterine Device
LARC: Long Acting Reversible Contraception
MSM: Men who have Sex with Men
PEP: Post-Exposure Prophylaxis (for prevention of HIV infection following possible HIV exposure)
PIS: Participant Information Sheet
PPE: Personal Protective Equipment - Equipment designed to protect the wearer from injury or infection; in this report, PPE is used to refer to equipment for protection from COVID-19 infection
PrEP: Pre-Exposure Prophylaxis (for prevention of HIV infection among people who are at risk of HIV exposure)
RCOG: Royal College of Obstetricians and Gynaecologists
SHA: Sexual Health Adviser
SRH: Sexual and Reproductive Health
STI: Sexually Transmitted Infection

## Footnotes

1 Health is a devolved matter in the UK. The first national lockdown was UK−wide but further lockdowns/restrictions were specific to different UK nations. We focus on the situation in England as this is where our study took place.

2 This paper focuses only on a sexual health and contraception service. Other SRH services included within the study in England were an abortion service and a sexual assault referral centre.

3 Sexual health advisers work in Sexual Health Clinics in the UK and Ireland. The role of SHA varies widely but generally involves working with individuals and groups affected by issues related to sexual health in general and STIs: see SSHA − Society of Sexual Health Advisers.

4 Due to frequent overlap in care seeking for sexual health and/or contraception e.g. STI screening prior to IUD fitting, results for contraception and sexual health will be combined.

5 Clinically extremely vulnerable refers to individuals who, due to specific health conditions, predispositions, or malignancy, are at a heightened risk of more severe manifestations of COVID−19 and death.
